# Accelerated Stack-of-Spirals Free-Breathing Three-Dimensional Ultrashort Echo Time Lung Magnetic Resonance Imaging: A Feasibility Study in Patients With Breast Cancer

**DOI:** 10.3389/fonc.2021.746059

**Published:** 2021-10-07

**Authors:** Min Jae Cha, Hye Shin Ahn, Hyewon Choi, Hyun Jeong Park, Thomas Benkert, Josef Pfeuffer, Mun Young Paek

**Affiliations:** ^1^ Department of Radiology, Chung-Ang University Hospital, Chung-Ang University College of Medicine, Seoul, South Korea; ^2^ Siemens Healthcare, Erlangen, Germany; ^3^ Siemens Healthineers Ltd., Seoul, South Korea

**Keywords:** lung, Magnetic Resonance Imaging, ultrashort echo time, acceleration, metastasis

## Abstract

**Purpose:**

To investigate the clinical feasibility of accelerated free-breathing stack-of-spirals (spiral) three-dimensional (3D) ultrashort echo time (UTE) lung magnetic resonance imaging (MRI) using iterative self‐consistent parallel imaging reconstruction from arbitrary k‐space (SPIRiT) algorithm in patients with breast cancer.

**Methods:**

The institutional review board approved this prospective study and patients’ informed consents were obtained. Between June and August 2018, 29 female patients with breast cancer underwent 3-T MRI including accelerated free-breathing spiral 3D UTE (0.98-mm isotropic spatial resolution; echo time, 0.05 msec) of the lungs and thin-section chest computed tomography (CT). Two radiologists evaluated the image quality and pulmonary nodules on MRI were assessed and compared, CT as a reference.

**Results:**

The pulmonary vessels and bronchi were visible consistently up to the sub-sub-segmental and sub-segmental branch levels, respectively, on accelerated spiral 3D UTE. The overall image quality was evaluated as good and excellent for 70.7% of accelerated spiral 3D UTE images (reviewer [R]1, 72.4% [21/29]; R2, 69.0% [20/29]) and acceptable for 20.7% (both R1 and R2, 20.7% [6/29]). Five patients on CT revealed 141 pulmonary metastatic nodules (5.3 ± 2.6 mm); the overall nodule detection rate of accelerated spiral 3D UTE was sensitivity of 90.8% (128/141), accuracy of 87.7%, and positive predictive value of 96.2%. In the Bland-Altman plot analysis comparing nodule size between CT and MRI, 132/141 nodules (93.6%) were inside the limits of agreement.

**Conclusion:**

Accelerated free-breathing spiral 3D UTE using the SPIRiT algorithm could be a potential alternative to CT for oncology patients.

## Introduction

With advances in magnetic resonance imaging (MRI), it has become possible to capture rapidly decaying signals of lung tissue, which have very short T2 and T2* values (1). Ultrashort echo time (UTE) imaging, offering an echo time (TE) < 1 ms, has emerged as a promising technique for lung MRI, enabling early acquisition of free induction decay at the end of the radiofrequency (RF) pulse (2, 3). Although the introduction of low-dose chest computed tomography (CT) has enabled a reduction of radiation dose, repeated CT examinations with cumulative radiation exposure may be harmful for patients who require longitudinal follow-up, such as patients with cancer.

The benefit of pulmonary MRI over CT as a radiation-free modality is desirable. However, achieving lung MRI with comparable diagnostic value to CT is challenging because of intrinsic short T2 and T2* properties of the lung and cardiac pulsation and respiratory motion artifacts. In addition, the acquisition time for pulmonary MRI is far longer than that for chest CT, accentuating the inefficacy of lung MRI.

To overcome these challenges, new techniques have been developed to improve the image quality and scan acceleration. One approach is the k-space sampling method. The two main k-space samplings of pulmonary UTE imaging consist of a radial acquisition into a sphere and a stack-of-spirals (spiral) acquisition into a cylinder (4–10). Several recent studies have demonstrated that spiral three-dimensional (3D) UTE enables high-resolution morphological imaging of the lungs with free-breathing and is more robust to motion with a shorter scan time than radial acquisition (7, 8). For the acceleration of non-Cartesian scans, an approach called SPIRiT (iterative self-consistent parallel imaging reconstruction) was proposed, which showed promising results while decreasing the acquisition time (11, 12).

In this study, we hypothesized that the combined use of spiral acquisition of 3D UTE with SPIRiT reconstruction can provide high-resolution pulmonary MR imaging with a reasonable scan time while free-breathing. In addition, it may be of great benefit for patients with cancer, if lung MRI enables detection of pulmonary metastasis as efficiently as thin-section CT, without radiation exposure. Therefore, the goal of this study was to determine the feasibility of accelerated free-breathing spiral 3D UTE in patients with breast cancer by assessing the image quality, scan time and diagnostic accuracy for the detection of pulmonary metastasis.

## Material and Methods

The Institutional Review Board approved this prospective study (IRB-1805-005-00326), and written informed consent was obtained at the time of the MRI.

### Study Population

**Figure 1:**
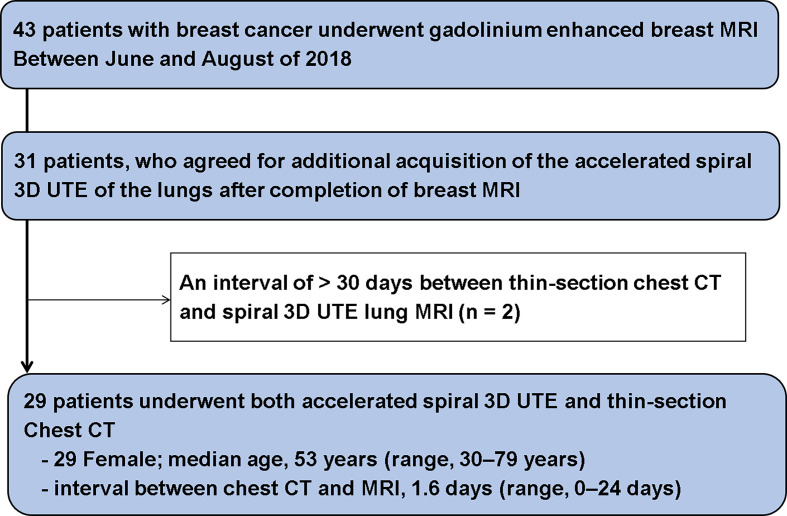
Flow chart of patient enrollment.

Between June and August 2018, 43 patients with breast cancer underwent gadolinium- enhanced breast MRI at our institution, for either preoperative evaluation of breast cancer or postoperative evaluation of recurrent tumor. Among them, we enrolled 31 patients who had agreed to undergo additional acquisition of accelerated spiral 3D UTE of the lungs after completion of breast MRI. During the analysis, two patients with an interval of > 30 days between thin-section chest CT and accelerated spiral 3D UTE MRI of the lungs, were excluded. Eventually, 29 female patients who underwent both, accelerated spiral 3D UTE examination of the lungs and thin-section chest CT, were included (**Figure 1**).

### MRI Protocols and Accelerated Spiral 3D UTE With SPIRiT Sequence

All participants underwent MRI on a 3-T MRI system (MAGNETOM Skyra, Siemens Healthcare, Erlangen, Germany) with a 30-channel anterior body coil in combination with a 32-channel spine matrix coil. All MRI examinations were performed with intravenous gadobutrol contrast administration (Gadovist; Bayer Healthcare, Berlin, Germany) at a dose of 0.1 mmol/kg into an ante-cubital vein. The prototypical spiral 3D UTE VIBE sequence with SPIRiT reconstruction was included as the last scan of each breast MRI. After acquisition of breast MRI in prone position, patients were placed in the supine position with their arms raised to avoid fold-in artifacts in UTE images. UTE sequences were acquired with a delay of > 10 min after contrast injection.

Due to the non-selective acquisition, UTE imaging was performed in coronal orientation to cover the entire lungs while avoiding infolding artifacts. The parameters of the spiral 3D UTE sequence were as follows: repetition time, 3.8 msec; minimum TE, 0.05 msec; field-of-view, 380 × 380 mm^2^; voxel size, 0.98 × 0.98 × 0.98 mm^3^; flip angle, 5°; RF pulse duration, 60 μs; number of slices, 176–208 depending on the patient volume. The TE varies with partition number and the time increases with distance from the center partition. Therefore, the minimum TE is equal to the time when the central k- space partition is acquired. The number of spirals used for in-plane encoding was 384. An in-plane under-sampling factor of R = 2 was used, resulting in 192 acquired spiral interleaves per 3D partition encoding. The spirals were designed to acquire the k-space center region denser than the k-space periphery.

For image reconstruction, a SPIRiT algorithm was used, which is an autocalibrating, coil-by-coil parallel reconstruction technique, based on self-consistency with the calibration and acquisition data (11). With SPIRIT, data from arbitrary k-space sampling patterns can be reconstructed. Duration of the spiral readout was 1280 µsec to reduce the susceptibility effects and T2* blurring at 3T and each spiral readout started in the center of k-space. To improve robustness to breathing motion, all k_z_ partitions were acquired per spiral interleaf before acquiring subsequent interleaves. Furthermore, prospective respiratory gating was used. For this, additional 1D projections were acquired throughout the sequence which were processed based on the manual selection of the coil element closest to the diaphragm edge. The scan was automatically stopped when sufficient data in the expiratory state was acquired, resulting in an average acquisition time of 225 s (range, 200–310 s), depending on the breathing pattern of the respective patients.

### Chest CT Acquisition

Enhanced chest CT images were obtained using a 64-detector row (Brilliance; Philips Healthcare, The Netherlands), 128-detector row (Optima 660; GE Healthcare, Waukesha, WI, USA), or 256-detector row (iCT; Philips Healthcare, The Netherlands) CT scanner using the following parameters: 120 kVp; 150–250 mA; beam pitch, 0.875–1.675; and section thickness, 1.3 mm for transverse images. Enhanced CT scanning was obtained 90 s after administration of contrast material (iopamidol: Pamiray 300; Dongkook Pharm, Seoul, Korea) at a rate of 1.5 mL/s with a power injector, followed by a 20 cc saline flushing at a rate of 1.5 mL/s. All imaging data were reconstructed with both, soft-tissue algorithm for mediastinal window images and bone algorithm for lung window images.

### Image Analysis

Original 3D coronal image data were reformatted into axial images and transferred to a commercially available picture archiving and communication system (Maroview version 5.4, MAROTECH Inc., Seoul, Korea).

#### Semiquantitative Assessment of Image Quality

Two board-certified radiologists, with 8 and 11 years of MRI experience, independently reviewed accelerated spiral 3D UTE images of the lungs. The pulmonary vasculature and airway depiction were rated using a five-point scoring system as follows: 1, main trunk level; 2, lobar level; 3, segmental branch level; 4, sub-segmental branch level; and 5, sub-sub-segmental branch level to the distal lung periphery. Image artifacts were rated as follows: 1, no lung structure was recognisable; 2, important artifacts; 3, moderate artifacts; 4, slight artifacts; and 5, no artifact. The overall image quality and mediastinal evaluation were scored as follows: 1, non-diagnostic; 2, poor; 3, acceptable; 4, good; and 5, excellent.

#### Assessment of Pulmonary Nodule Detection

Pulmonary nodules present as rounded or irregular opacities, over 2 mm in diameter (13). To provide a standard reference of pulmonary nodules, two board-certified radiologists with 5 and 8 years of CT experience reviewed the CT images in consensus. Both mediastinal (width, 400 HU; level, 20 HU) and lung (width, 1500 HU; level, −700 HU) window CT images were assessed. Lung parenchymal calcifications and fissural nodules on CT were not included in the analysis (14).

The assessment of each nodule on accelerated spiral 3D UTE imaging was repeated by two board-certified radiologists with 8 and 11 years of MRI experience, blinded to the CT results. Disagreements regarding nodule detection were resolved by consensus. The mean diameter measured by the two readers was used as the nodule size. We assessed the nodule’s location according to its axial, craniocaudal, and lobar locations. Regarding the axial location, the outer third of the lung was defined as peripheral and the inner third as central. All other nodules were considered to be located in the mid-lung (15). Lobar location was defined as one of the five lobes: the right upper, right middle, right lower, left upper, and left lower.

### Statistical Analysis

Continuous data are expressed as the mean and standard deviation; all categorical data are presented as proportions. In the semiquantitative analysis of image quality, inter-observer agreement was evaluated using kappa (k) analysis. The sensitivity, accuracy, and positive predictive value for detection of pulmonary metastasis on accelerated spiral 3D UTE, CT as a reference, were calculated. Inter-observer agreement for nodule size measurement on accelerated spiral 3D UTE was determined using intraclass correlation coefficient (ICC). The sizes of the pulmonary nodules measured on MRI and CT were visually demonstrated with Bland-Altman limits of agreement. P < 0.05 was considered significant. All statistical analyses were performed using IBM SPSS Statistics for Windows version 20.0 (IBM Corp., Armonk, NY, USA).

## Results

Accelerated spiral 3D UTE lung MR examinations were successfully performed without any adverse events in the entire study population (29 female patients with pathologically confirmed breast cancer; median age, 53 years; range, 30–79 years). The average acquisition time for free-breathing spiral 3D UTE with SPIRiT was 225 s (range, 200–310 s), depending on patient’s breathing pattern. The average time for image reconstruction was 276 s (range, 210–337 s), depending on the size of imaging data.

### Semiquantitative Assessment of Image Quality

**Table 1** demonstrates the visual scores for semiquantitative image quality analysis for intrapulmonary structures. Pulmonary vessels were visible consistently up to the sub-sub-segmental (reviewer 1 [R1], 89.7% [26/29]; reviewer 2 [R2], 82.8% [24/29]) or sub-segmental (R1, 10.3% [3/29]; R2, 17.2% [5/29]) levels on accelerated spiral 3D UTE. Both readers reported that airways were visible up to the sub-segmental (R1, 82.8% [24/29]; R2, 79.3% [23/29]) or segmental (R1, 17.2% [5/29]; R2, 20.7% [6/29]) bronchi. For mediastinal evaluation, accelerated spiral 3D UTE images were rated as acceptable for 34.5% of UTE images (both R1 and R2, 34.5% [10/29]), good for 46.6% (R1, 44.8% [13/29]; R2, 48.3% [14/29]), and excellent for 19% (R1, 20.7% [6/29]; R2, 17.2% [5/29]). Both readers noted slight image artifacts in 51.7% (15/29) of UTE images, whereas important or moderate artifacts that may deteriorate image quality were recorded in 48.3% (14/29) images. The overall image quality was evaluated as good and excellent for 70.7% of UTE images (R1, 72.4% [21/29]; R2, 69.0% [20/29]), acceptable for 20.7% (both R1 and R2, 20.7% [6/29]), and poor for 8.6% (R1, 7.4% [2/29]; R2, 10.3% [3/29]).

**Table 1:** Semiquantitative analysis of the image quality of accelerated spiral 3D UTE sequence in 29 patients with breast cancer.

Parameters		Visual Scoring					Mean	Kappa	P value
		1	2	3	4	5			
Pulmonary vascular depiction	Reader 1	0	0	0	3	26	4.86 ± 0.35	0.760	< 0.001
	Reader 2	0	0	0	5	24	4.79 ± 0.41
Airway depiction	Reader 1	0	0	5	24	0	3.83 ± 0.38	0.664	< 0.001
	Reader 2	0	0	6	23	0	3.79 ± 0.41
Mediastinal evaluation	Reader 1	0	0	10	13	6	3.86 ± 0.74	0.836	< 0.001
	Reader 2	0	0	10	14	5	3.83 ± 0.71
Image artifacts	Reader 1	0	1	13	15	0	3.48 ± 0.57	0.683	< 0.001
	Reader 2	0	2	12	15	0	3.45 ± 0.63
Overall Image quality	Reader 1	0	2	6	14	7	3.90 ± 0.86	0.838	< 0.001
	Reader 2	0	3	6	14	6	3.78 ± 0.93

UTE, ultrashort echo time.

All inter-observer agreements for visual scoring were statistically significant (P < 0.001). They were assessed as good for pulmonary vascular and airway depiction and image artifacts (k = 0.760, 0.664, and 0.683, respectively) and excellent for mediastinal evaluation and overall image quality (k = 0.836 and 0.838, respectively).

### Assessment of Pulmonary Nodule Detection

**Figure 2:**
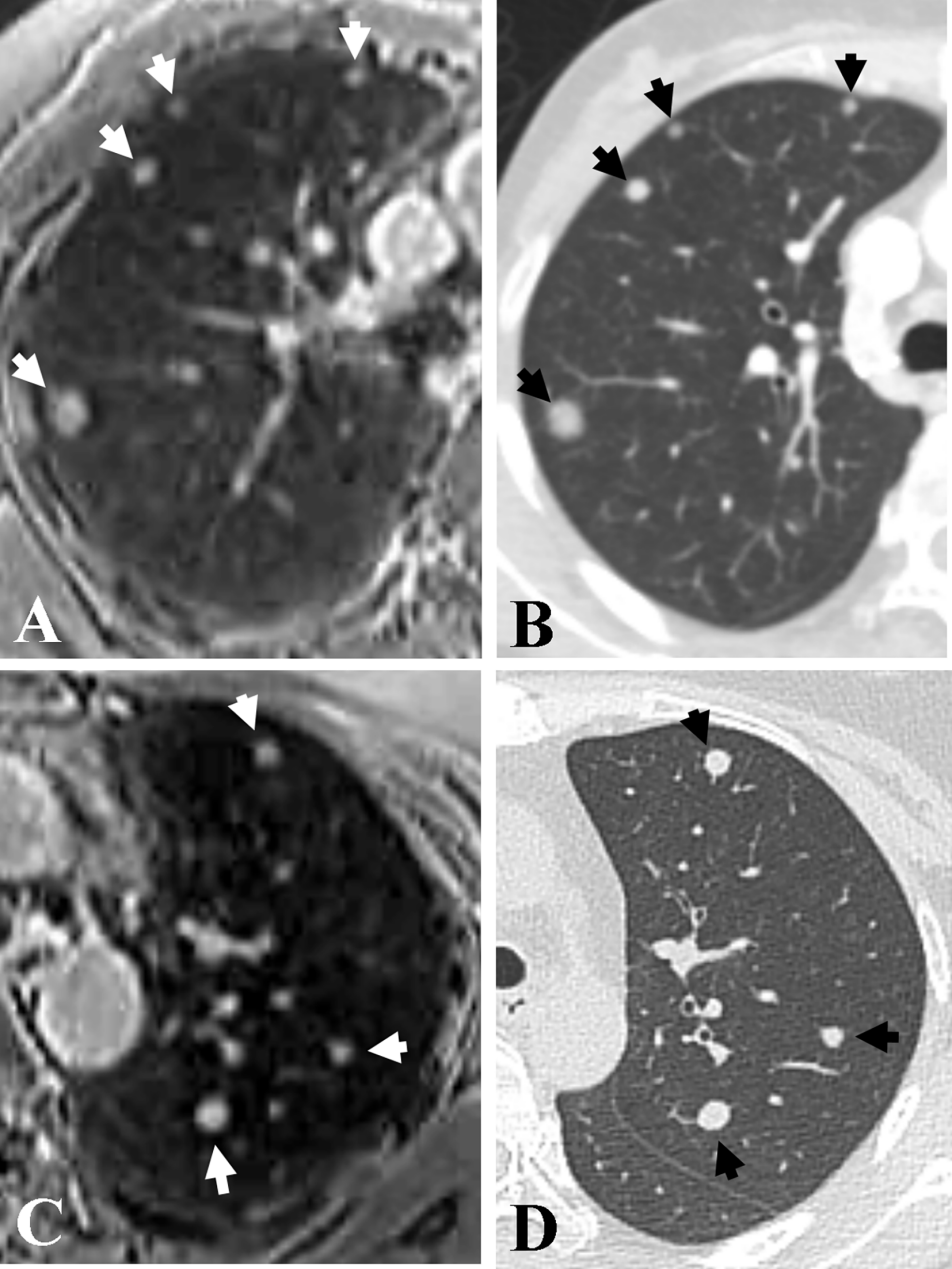
Representative axial images of accelerated spiral 3D UTE **(A, C)** and CT **(B, D)** for the detection of pulmonary metastasis. Seven nodules (arrows; range, 3.5 mm–10.6 mm in diameter) are detected in both upper lobes in a 38-year-old patient with bilateral breast cancer. The overall image quality rating for spiral 3D UTE images **(A, C)** was excellent.

A total of 141 pulmonary metastatic nodules were identified in five patients using thin-section CT. The mean interval between chest CT and MRI was 1.6 days (range, 0–24 days). The mean dose-length product for chest CT was 262.5 ± 72.2 mGy·cm and the mean effective dose was 3.7 ± 1.0 mSv. The detection rates of pulmonary nodules according to size and location are summarized in (**Table 2** and **Figure 2**). Of the 141 nodules detected on CT, 128 were also detected on the accelerated spiral 3D UTE, with a nodule detection rate of 90.8%. Five nodules (three nodules in right lower lobe and two nodules in left upper lobe; mean size, 3.2 mm) on MRI was not depicted on the reference CT scan and was considered a false-positive result. The overall diagnostic performance of accelerated spiral 3D UTE for pulmonary nodule detection was as follows: sensitivity, 90.8%; accuracy, 87.7%; and positive predictive value, 96.2%. All 28 nodules ≥ 7 mm in diameter were identified on spiral 3D UTE images (100%). Among the 113 nodules < 7 mm in diameter, 100 nodules were detected on MRI, showing a detection rate of 88.5%. The detection rate was lower for central lung nodules (71.4%) than for peripheral lung nodules (91.7%). In terms of craniocaudal distribution, the detection rate was lower for nodules in both lower lobes (37/43, 86.1%) than for nodules in both upper lobes (78/85, 91.8%).

**Table 2:** Characteristics and detection rate of pulmonary nodules.

Parameters	Accelerated Spiral 3D UTE with SPIRiT	CT (reference)	Detection rate
No. of overall nodules detected	128	141	90.8%
Mean nodule diameter (mm)	5.15 ± 2.56	5.31 ± 2.64	
No. of nodules; of given diameter			
2 mm ≤ diameter < 5 mm	74	84	88.1%
5 mm ≤ diameter < 7 mm	26	29	89.7%
7 mm ≤ diameter < 10 mm	24	24	100%
10 mm ≤ diameter	4	4	100%
No. of nodules; per axial location			
Central	5	7	71.4%
Mid-lung	24	26	92.3%
Peripheral	99	108	91.7%
No. of nodules; per lobar location			
Right upper lobe	28	31	90.3%
Right middle lobe	13	13	100%
Right lower lobe	18	21	85.7%
Left upper lobe	50	54	92.6%
Left lower lobe	19	22	86.4%

3D, three-dimensional; UTE, ultrashort echo time; SPIRiT, Iterative self‐consistent parallel imaging reconstruction from arbitrary k‐space; CT, computed tomography.

### Nodule Size According to CT and MRI Measurements

**Figure 3:**
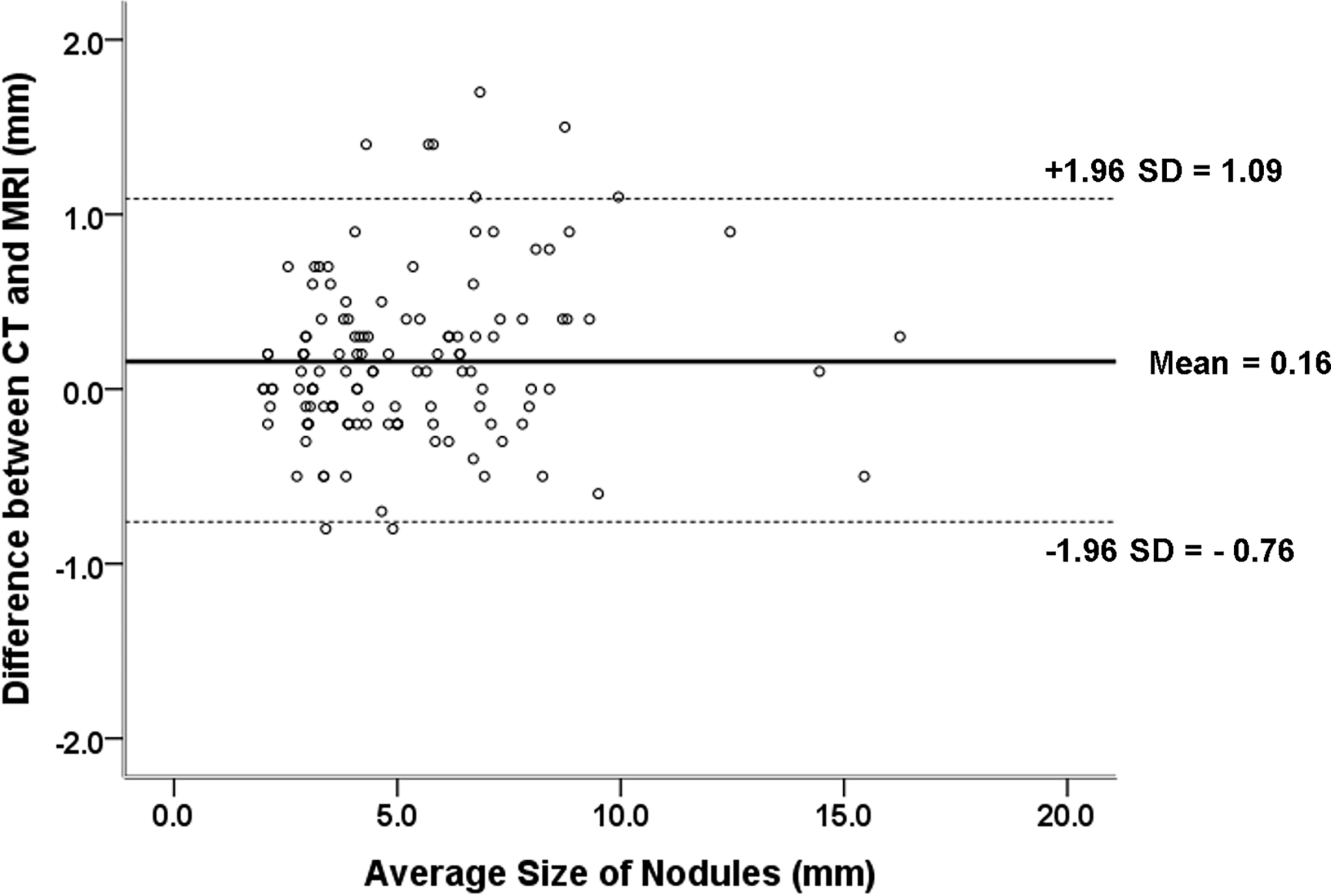
Difference between pulmonary nodule size measurements on CT and MRI against the mean measurement with 95% limits of agreement (mean difference between CT and MRI measurements, 0.16 mm; 95% limits of agreement, –0.76 mm and 1.09 mm).

**Figure 4:**
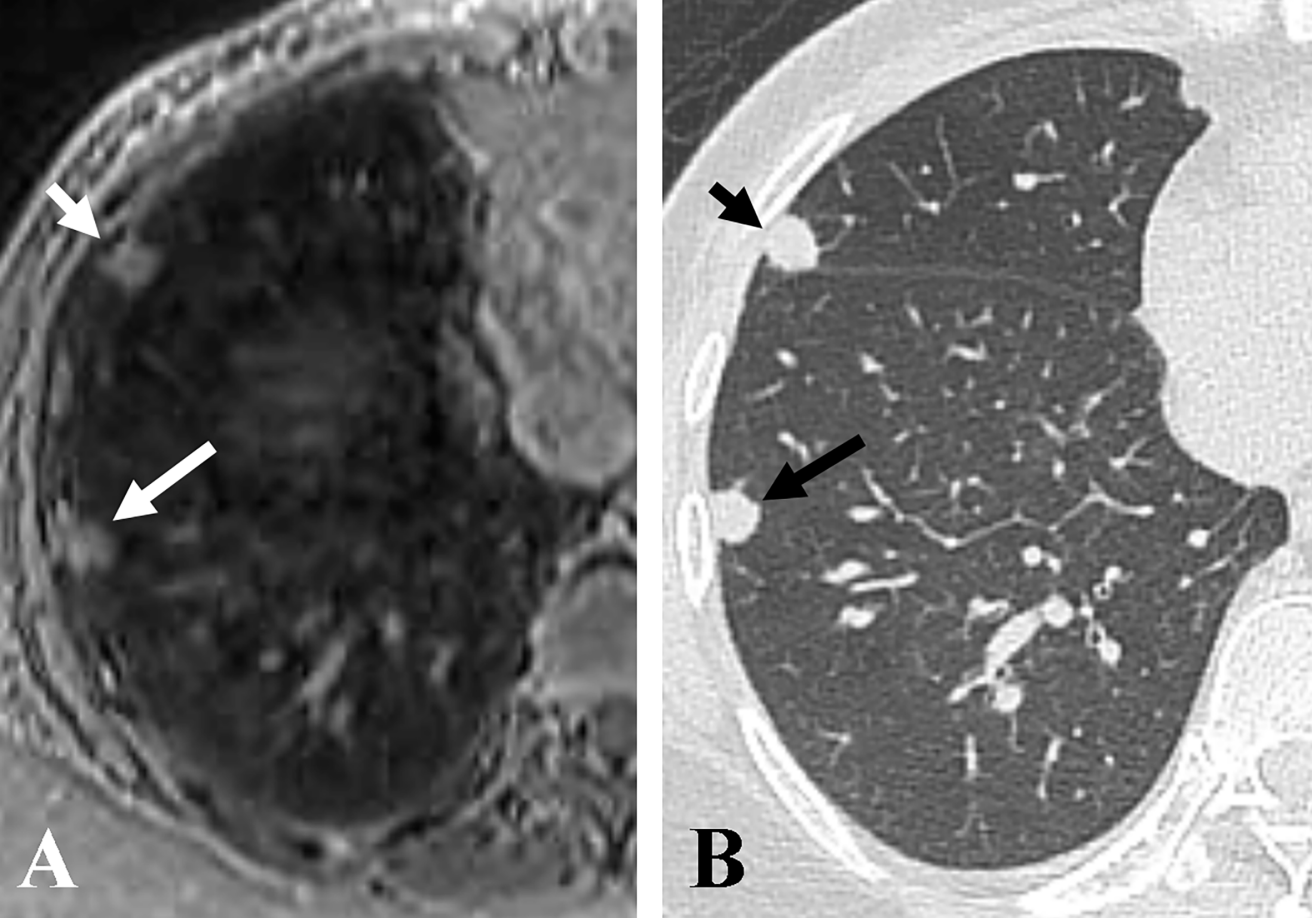
Representative axial images of accelerated spiral 3D UTE **(A)** and CT **(B)**, showing nodule size underestimation on MRI compared with CT for pleural-based nodules. The overall image quality rating for spiral 3D UTE image was good. The sizes of two pleural-based nodules in the right middle lobe (short arrows) and right lower lobe (long arrows) were 9.5 mm and 7.7 mm on CT, and 8 mm and 6.2 mm on accelerated spiral 3D UTE, respectively.

The nodule size measured on accelerated spiral 3D UTE (mean, 5.15 ± 2.56 mm; range, 0.2–16.1 mm) was slightly smaller than that on high-resolution CT (mean, 5.31 ± 2.64 mm; range, 0.2–16.4 mm; mean difference, 0.16 mm). In the Bland-Altman plot analysis, 9/141 nodules (6.38%) were outside the limits of agreement (**Figure 3**). All nodules outside the limits of agreement were specifically located in the subpleural (n = 8) and peri-fissural (n = 1) areas of the lungs (**Figure 4**).

The ICC for nodule size measurement on accelerated spiral 3D UTE between two observers was 0.994 (95% confidence interval, 0.987 - 0.997, P < 0.001).

## Discussion

The lung is one of the most common sites of distant metastasis in solid tumors (16). Thus, most patients with cancer should undergo regular chest CT to evaluate pulmonary metastasis with inevitable radiation exposure. Furthermore, the number of incidentally-detected or screening-detected pulmonary nodules, which need follow-up, has increased after the implementation of lung cancer screening with low-dose CT (17). Our results demonstrate that accelerated free-breathing spiral 3D UTE sequence can provide acceptable to good quality images of the lungs with sub-millimeter isotropic spatial resolution, showing a detection rate (sensitivity) of 90.8% for pulmonary metastasis in patients with breast cancer. This finding supports the hypothesis that accelerated free-breathing spiral 3D UTE may play a role in pulmonary metastasis workup, as an alternative to chest CT, thus avoiding radiation exposure.

Many previous studies have investigated lung MRI as an alternative to CT for pulmonary nodule detection (5, 8, 14, 18–20). Recently, UTE has emerged as a promising technique with high-resolution lung MRI showing a pulmonary nodule detection rate ranging from 73.2% to 93.0% (5, 8, 14, 20). In the current study, the overall nodule detection rate on accelerated spiral 3D UTE images was as high as 90.8%, and the sensitivity for lung nodules larger than 7 mm in diameter was 100%. In addition, the detection rate for nodules measuring 2-5 mm was 88.1%, which is higher than those in previous reports by Burris et al. (17% for nodules smaller than 4 mm in diameter; 1.3-mm isovoxel spatial resolution), Ohno et al. (74.1% for nodules of 4-6 mm in diameter; 1-mm isovoxel spatial resolution), and Cha et al. (76.7% for nodules 2-5 mm in diameter; 1.5-mm isovoxel spatial resolution) (5, 8, 14). Sub-millimetre-sized spatial resolution is a contributing factor in the progress of detecting smaller nodules. Another reason could be the application of stack-of-spirals trajectory in the current study, which has advantages of high k-space coverage speed with lower artifacts and respiratory synchronization (7).

Previously, Cha et al. demonstrated the feasibility of spiral 3D UTE with 1.5-mm isotropic spatial resolution to detect pulmonary nodules in oncology patients (8). One major advancement of the current study is that we applied undersampling of k-space in combination with SPIRiT reconstruction (10, 11). With SPIRiT implementation, mean image acquisition duration was reduced from 327 s to 225 s (31.2% of scan time reduction), while spatial resolution was improved from 1.5-mm to 1-mm isotropic voxel size. Moreover, the image acquisition time of accelerated spiral 3D UTE was far shorter than that of radial trajectory-based UTE, such as PETRA, which still needs respiratory-trigger using a belt or breath-hold (4, 7). Accelerated spiral 3D UTE with automatic respiratory synchronization enabled free-breathing image acquisition with shorter scan time (usually less than 4 minutes), which may enhance patient convenience and cost-effectiveness of lung MRI.

Regarding inter-modality difference in nodule size measurement, we found that pulmonary nodule diameter may be underestimated on MRI compared with CT, similar to prior reports. However, the measurement differences between CT and MRI in our study were much smaller (0.16 mm on average) compared with prior reports, in which inter-modality measurement differences were 1-4 mm in diameter (14, 21). Notably, in our study, the nine nodules that were outside the limits of agreement showed juxta-pleural location. A possible explanation could be a difficulty in identifying the accurate borderline for pleural-based or fissural-attached nodules. Many previous reports have demonstrated that juxta-pleural nodules have a risk of inter-reader and intra-reader volume variability due to indistinct nodule borders (22, 23).

There are several limitations to our study. First, it is a single-centre study with a relatively small study population. Second, it is difficult to apply our scan parameters for spiral 3D UTE with SPIRiT in other institutions. UTE sequences are sensitive to field inhomogeneities and gradient hardware, thus, modifications may be required. Third, all nodules were presumed to show metastasis, however, pathologic correlations were not available. Finally, readers of accelerated spiral 3D UTE in our study were two experienced radiologists with at least 8-year experience in MR imaging, thus pulmonary nodule detection rate could be overestimated. Inexperienced readers may not achieve a comparable high diagnostic performance.

## Conclusion

We demonstrated the clinical feasibility of accelerated spiral 3D UTE lung imaging of sub-millimeter-sized isotropic spatial resolution in patients with breast cancer. Accelerated spiral 3D UTE enabled free-breathing high-resolution lung imaging with a reasonable scan time of approximately 4 min, showing a high sensitivity for the detection of pulmonary nodules and acceptable image quality, thus providing a potential alternative to CT for patients who need regular imaging follow-up.

## Data Availability Statement

The original contributions presented in the study are included in the article/supplementary material. Further inquiries can be directed to the corresponding author.

## Ethics Statement

The studies involving human participants were reviewed and approved by the Institutional Review Board of Chung-Ang University Hospital. The patients/participants provided their written informed consent to participate in this study.

## Author Contributions

MJC: conceptualization, data curation, funding acquisition, investigation, methodology, visualization, and writing - original draft. HSA: conceptualization, data curation, formal analysis, investigation, methodology, and writing - review & editing. HC: investigation and formal analysis. HJP: conceptualization, supervision, and writing - review & editing. TB: software, validation, and writing - review & editing. JP: software and writing - review & editing. MYP: software, validation, and writing – review & editing. All authors contributed to the article and approved the submitted version.

## Funding

MJC received Chung-Ang University Research Grants in 2020.

## Conflict of Interest

TB, JP, and MYP are employed by Siemens Healthcare.

The remaining authors declare that the research was conducted in the absence of any commercial or financial relationships that could be construed as a potential conflict of interest.

## Publisher’s Note

All claims expressed in this article are solely those of the authors and do not necessarily represent those of their affiliated organizations, or those of the publisher, the editors and the reviewers. Any product that may be evaluated in this article, or claim that may be made by its manufacturer, is not guaranteed or endorsed by the publisher.
